# Supplementary material for: Peptidoglycan-tethered and free forms of the Braun lipoprotein are in dynamic equilibrium in Escherichia coli
Source: eLife. 2024 Oct 3;12:RP91598. doi: 10.7554/eLife.91598 (PMC11449479; doi:10.7554/eLife.91598)
Supplement: Supplementary file 1. — This file contains mass spectral data obtained for the kinetics analyses for the BW25113 strain and the ΔyafK mutant. [file elife-91598-supp1.pdf]

BW25113
t = 0 min

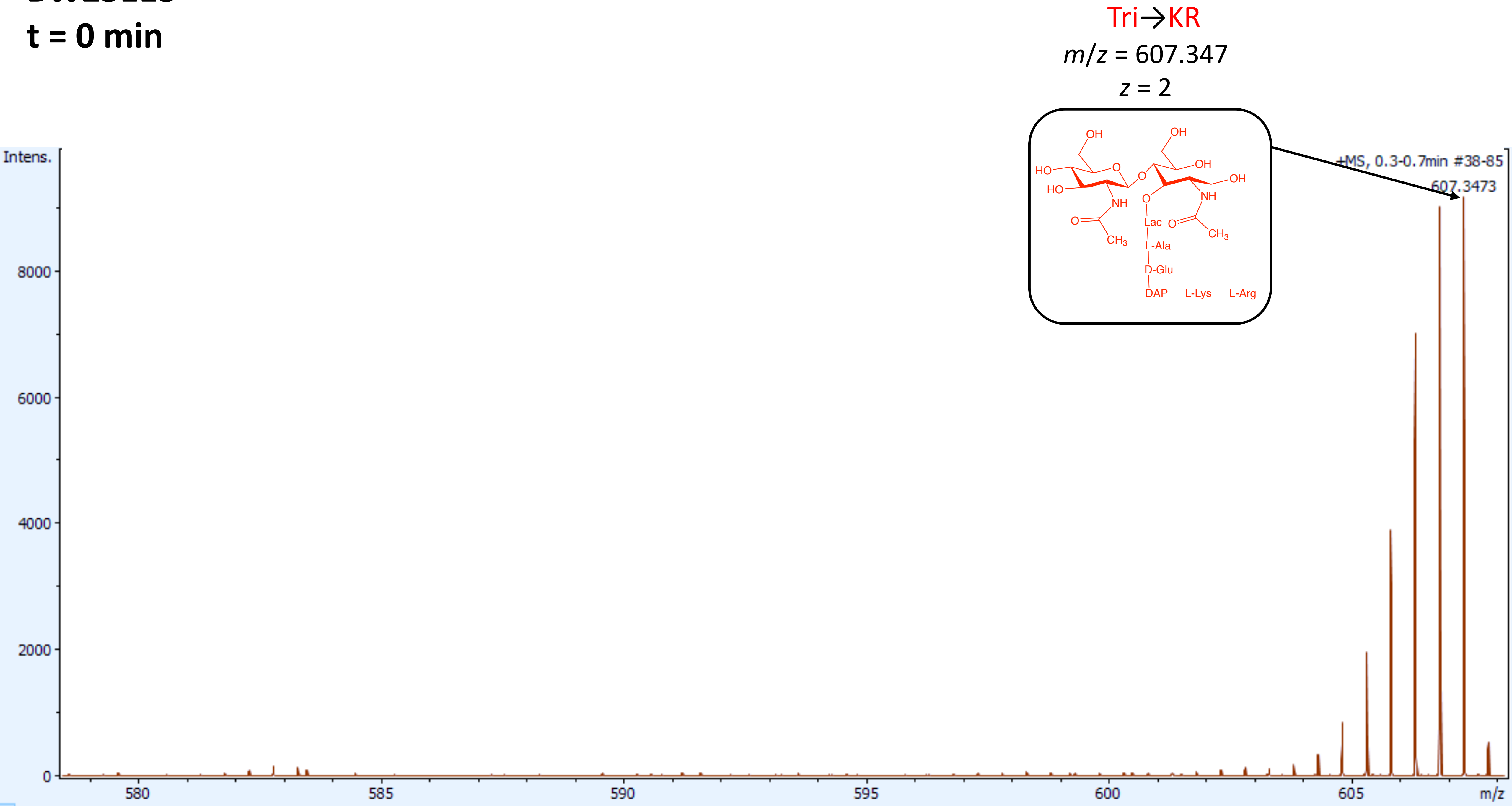

BW25113

t =5 min

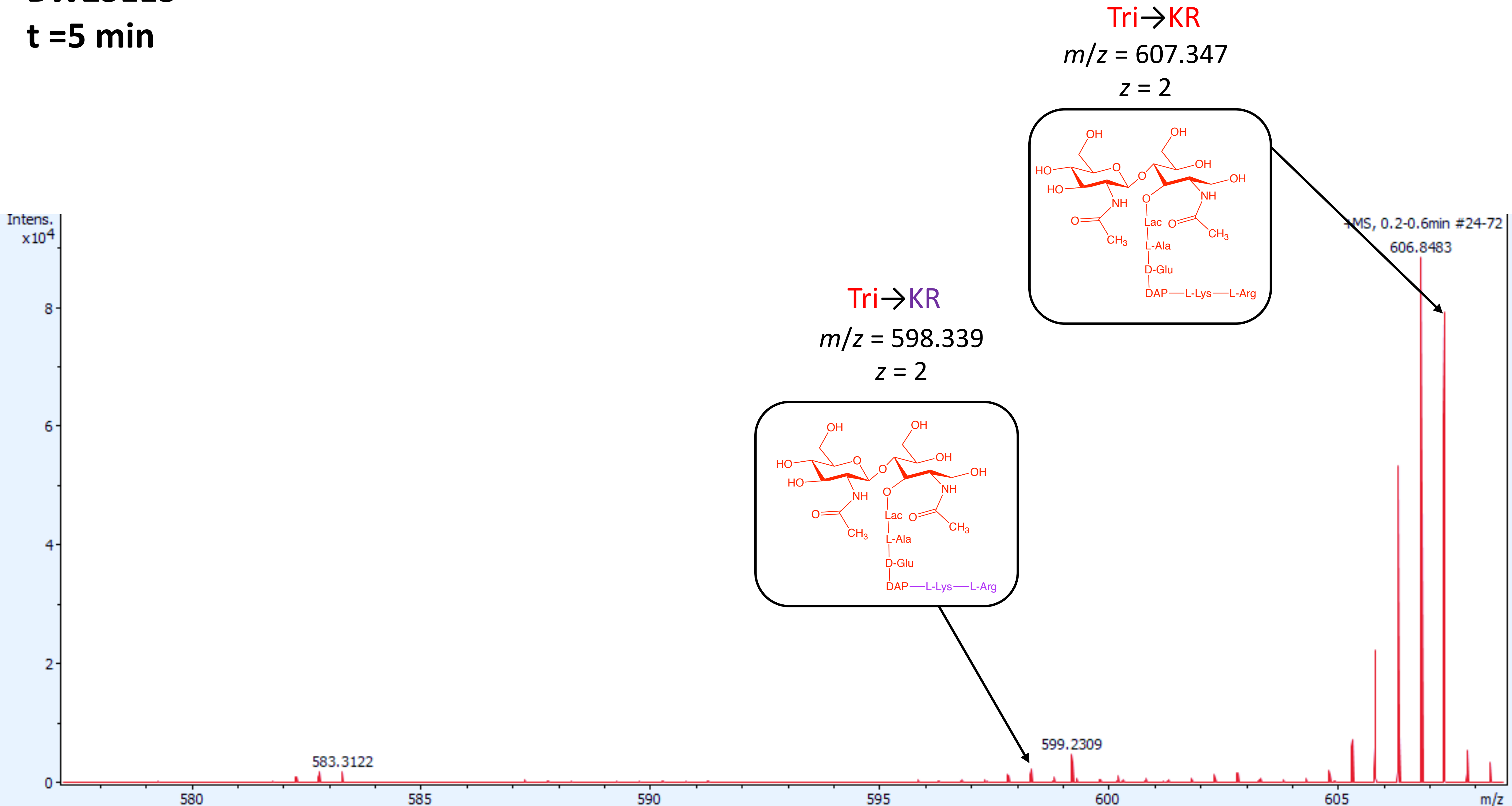

BW25113

t = 10 min

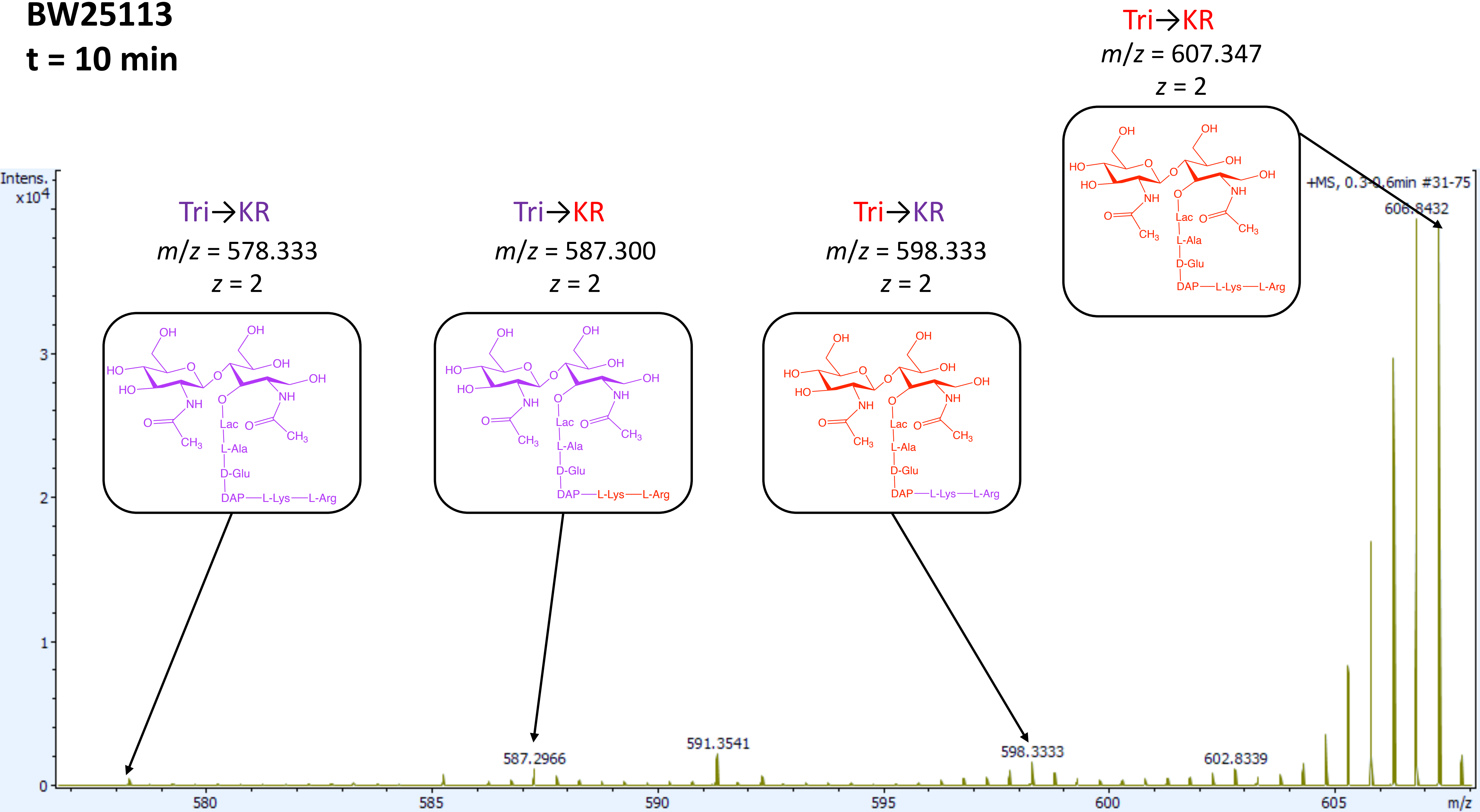

BW25113
t = 20 min

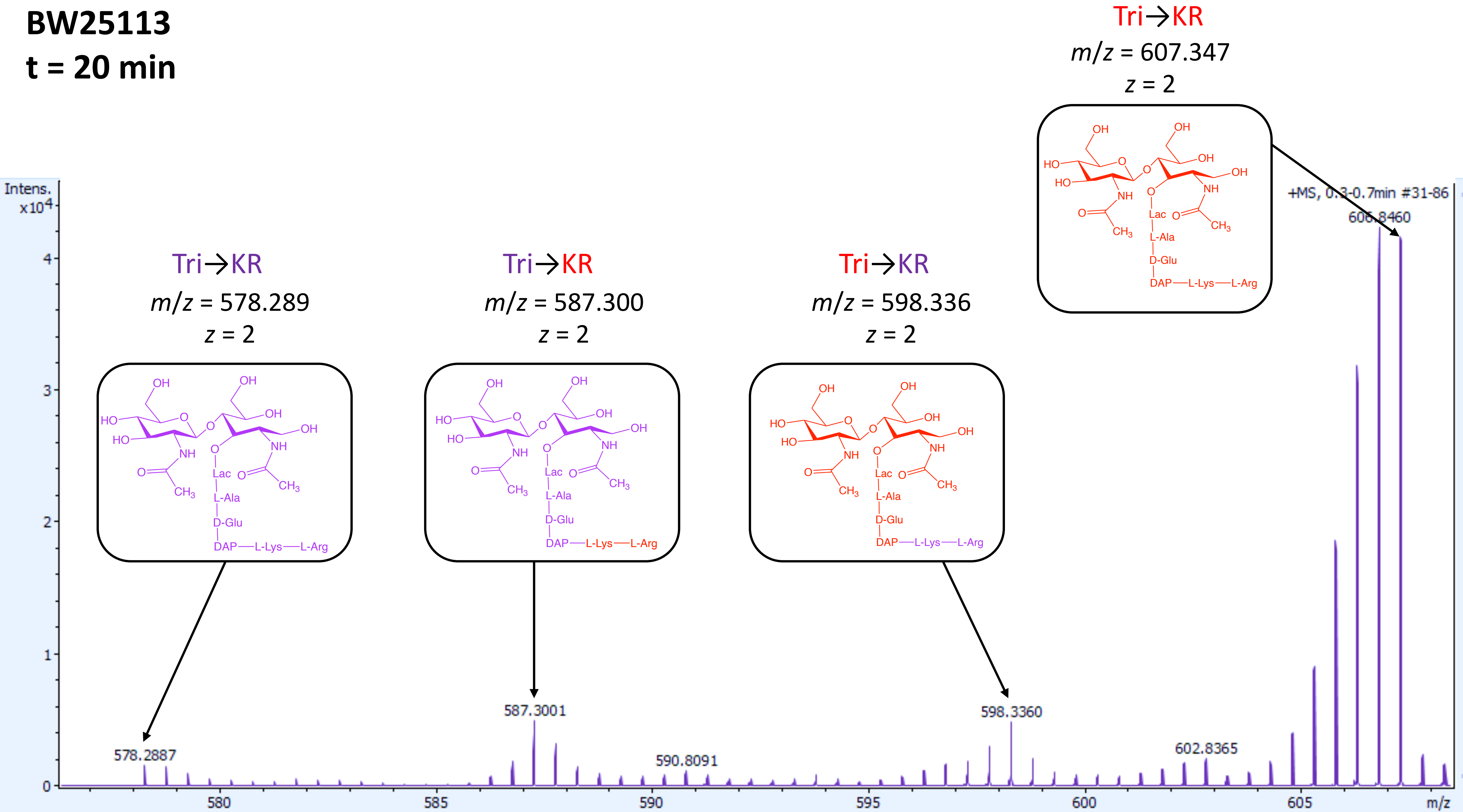

BW25113

t = 40 min

Tri→KR	Tri→KR	Tri→KR	Tri→KR
m/z = 578.289	*m/z* = 587.300	*m/z* = 598.336	*m/z* = 607.347
z = 2	*z* = 2	*z* = 2	*z* = 2

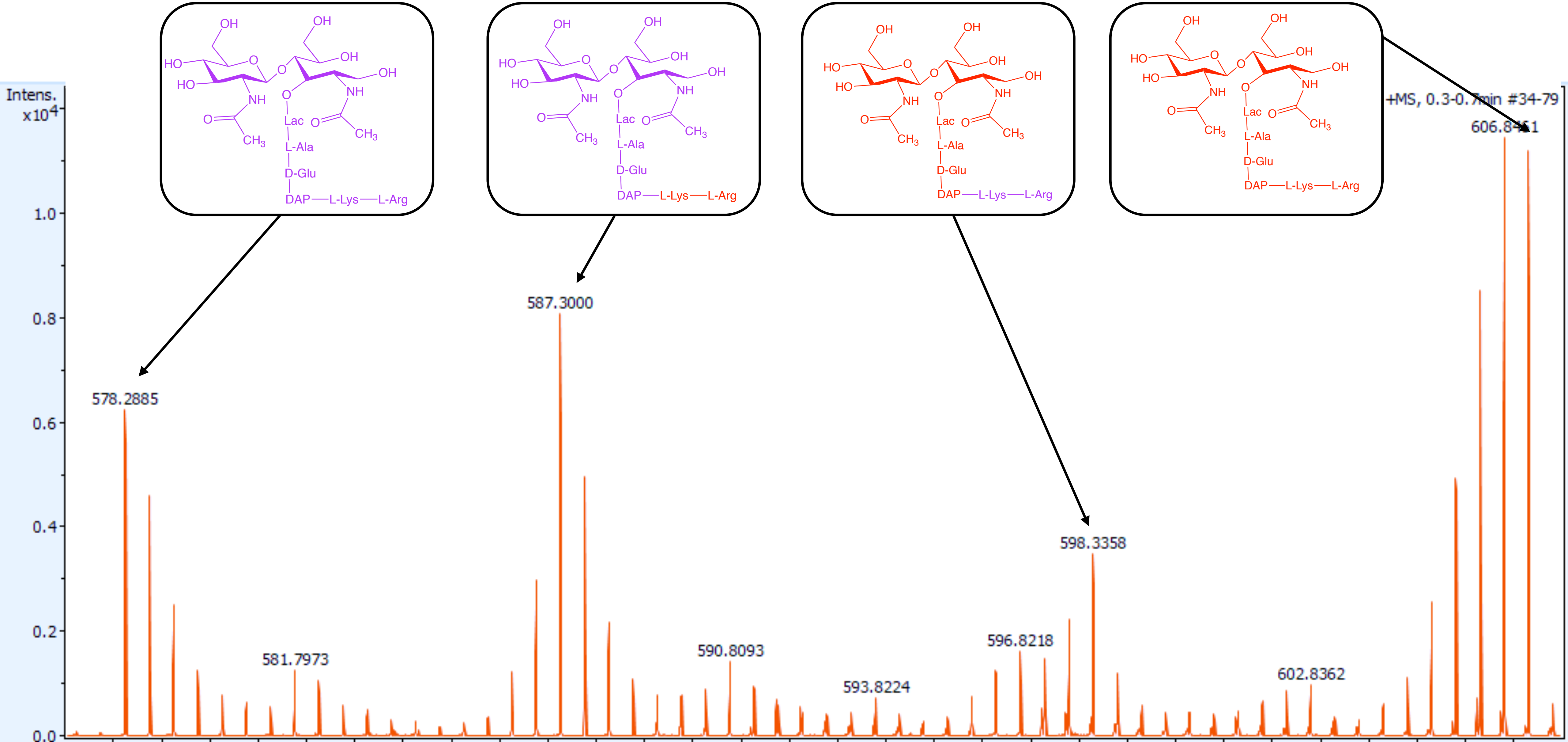

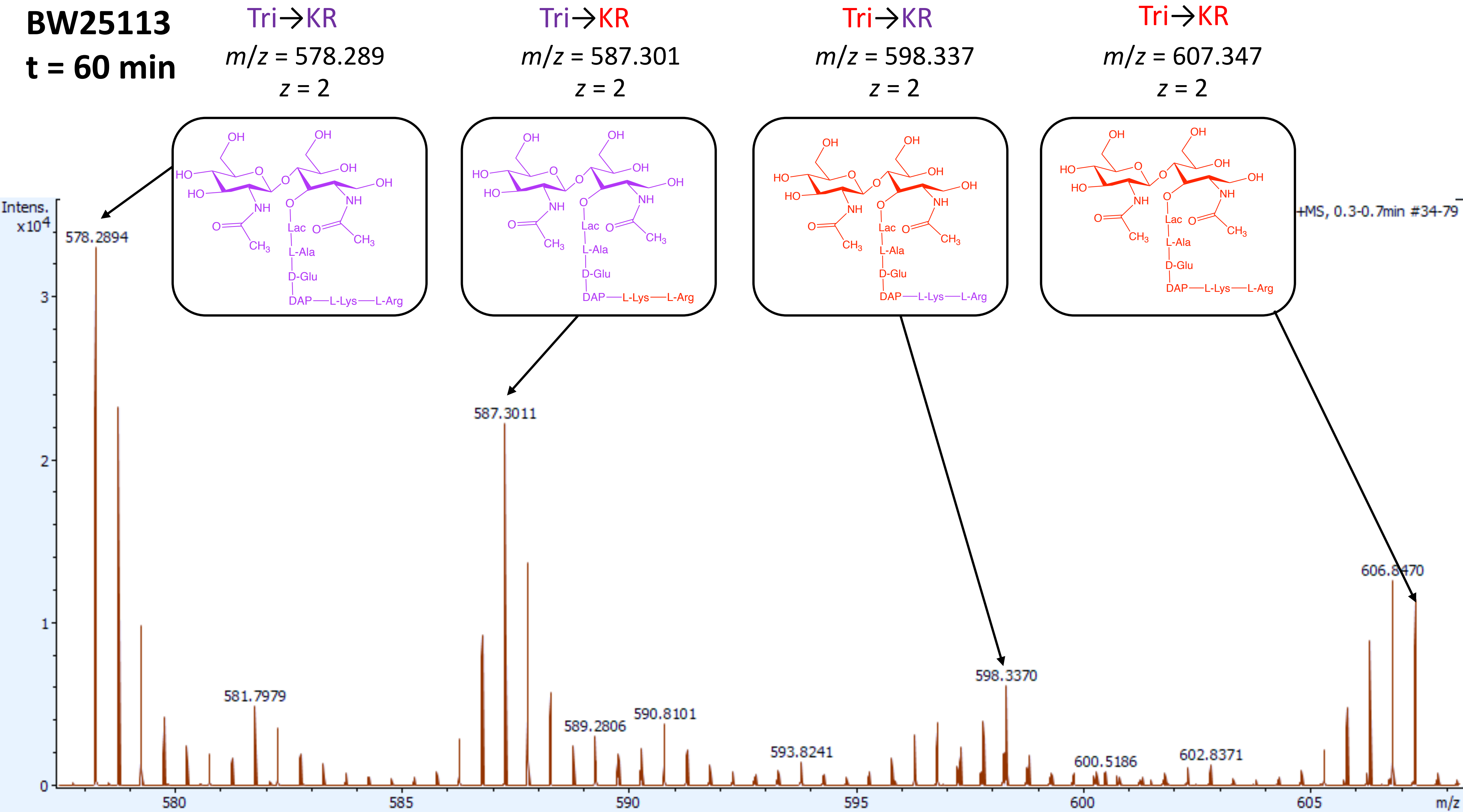

BW25113
t = 60 min
Tri→KR
m/z = 578.289
z = 2
Tri→KR
m/z = 587.301
z = 2
Tri→KR
m/z = 598.337
z = 2
Tri→KR
m/z = 607.347
z = 2
Lac
L-Ala
D-Glu
DAP—L-Lys—L-Arg
Intens.
x10^4
+MS, 0.3-0.7min #34-79
578.2894
581.7979
587.3011
589.2806
590.8101
593.8241
598.3370
600.5186
602.8371
606.8470
m/z

ΔyafK
t = 0 min

Tri→KR
m/z = 607.347
z = 2

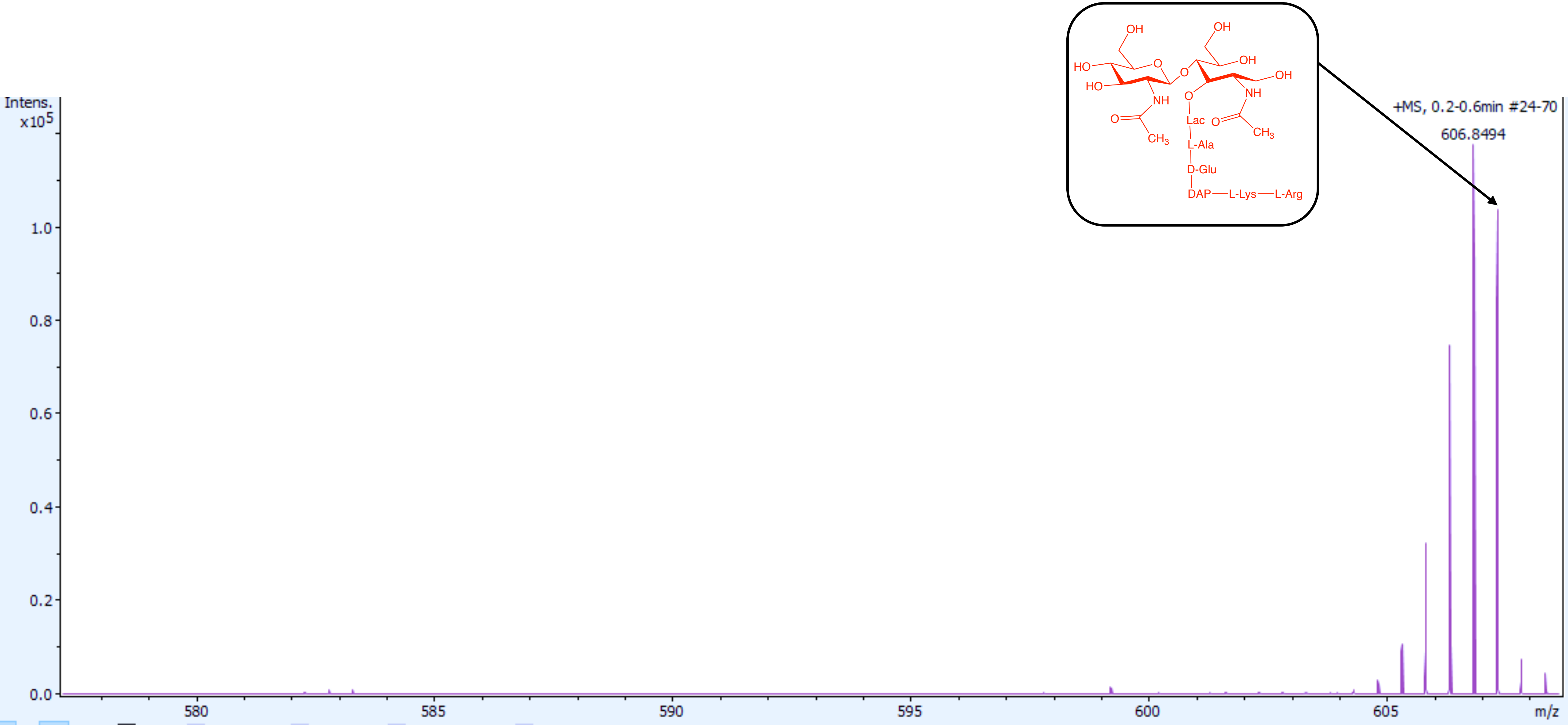

ΔyafK

t = 5 min

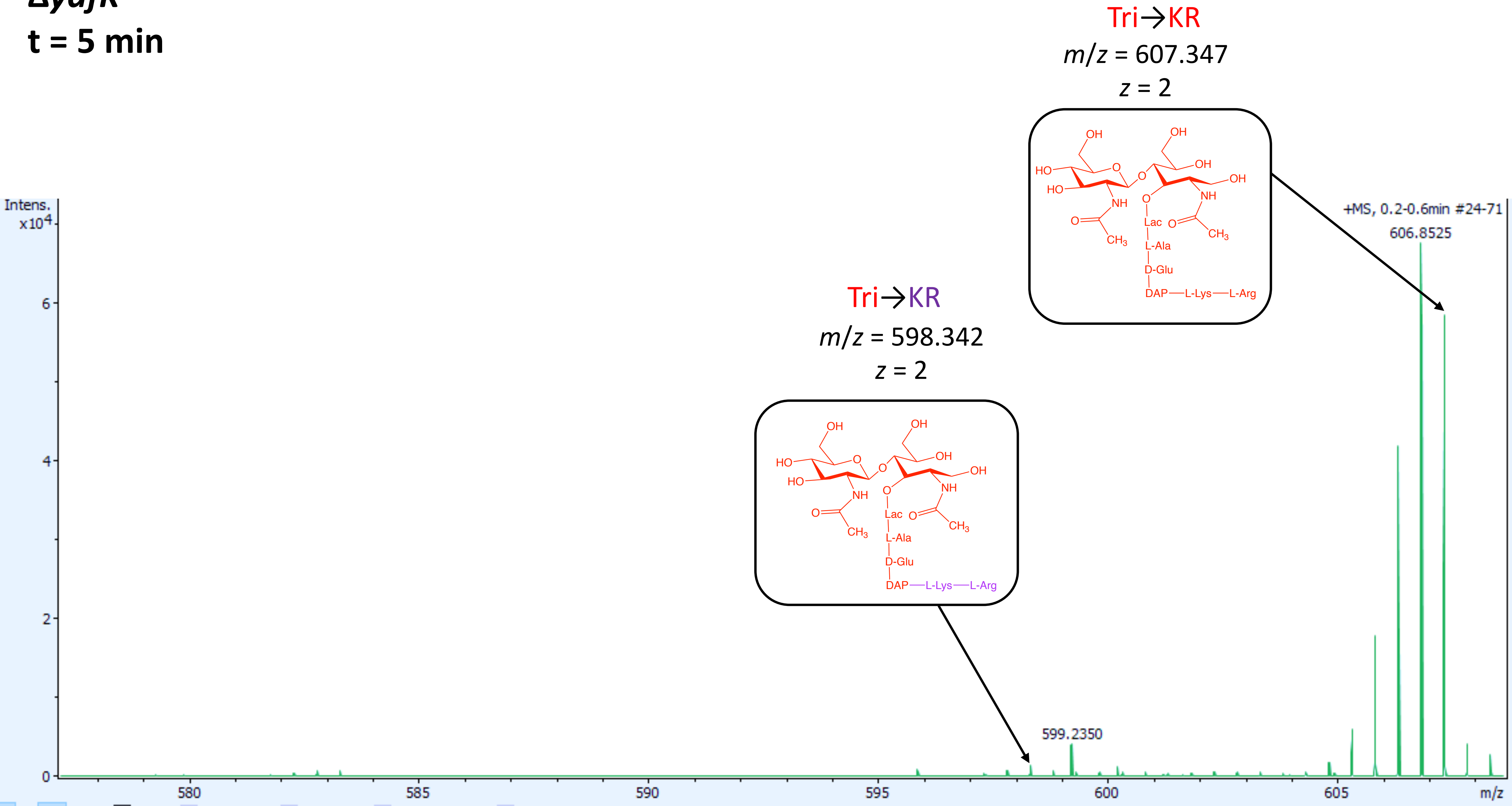
Tri→KR
m/z = 607.347
z = 2
OH
OH
HO
O
O
OH
OH
HO
NH
O
NH
O
Lac
O
CH3
CH3
L-Ala
D-Glu
DAP—L-Lys—L-Arg
Tri→KR
m/z = 598.342
z = 2
OH
OH
HO
O
O
OH
OH
HO
NH
O
NH
O
Lac
O
CH3
CH3
L-Ala
D-Glu
DAP—L-Lys—L-Arg
Intens.
x10^4
+MS, 0.2-0.6min #24-71
606.8525
599.2350
0
2
4
6
580
585
590
595
600
605
m/z

Δ*yafK*
t = 10 min

Tri→KR
m/z = 598.336
z = 2

Tri→KR
m/z = 607.346
z = 2

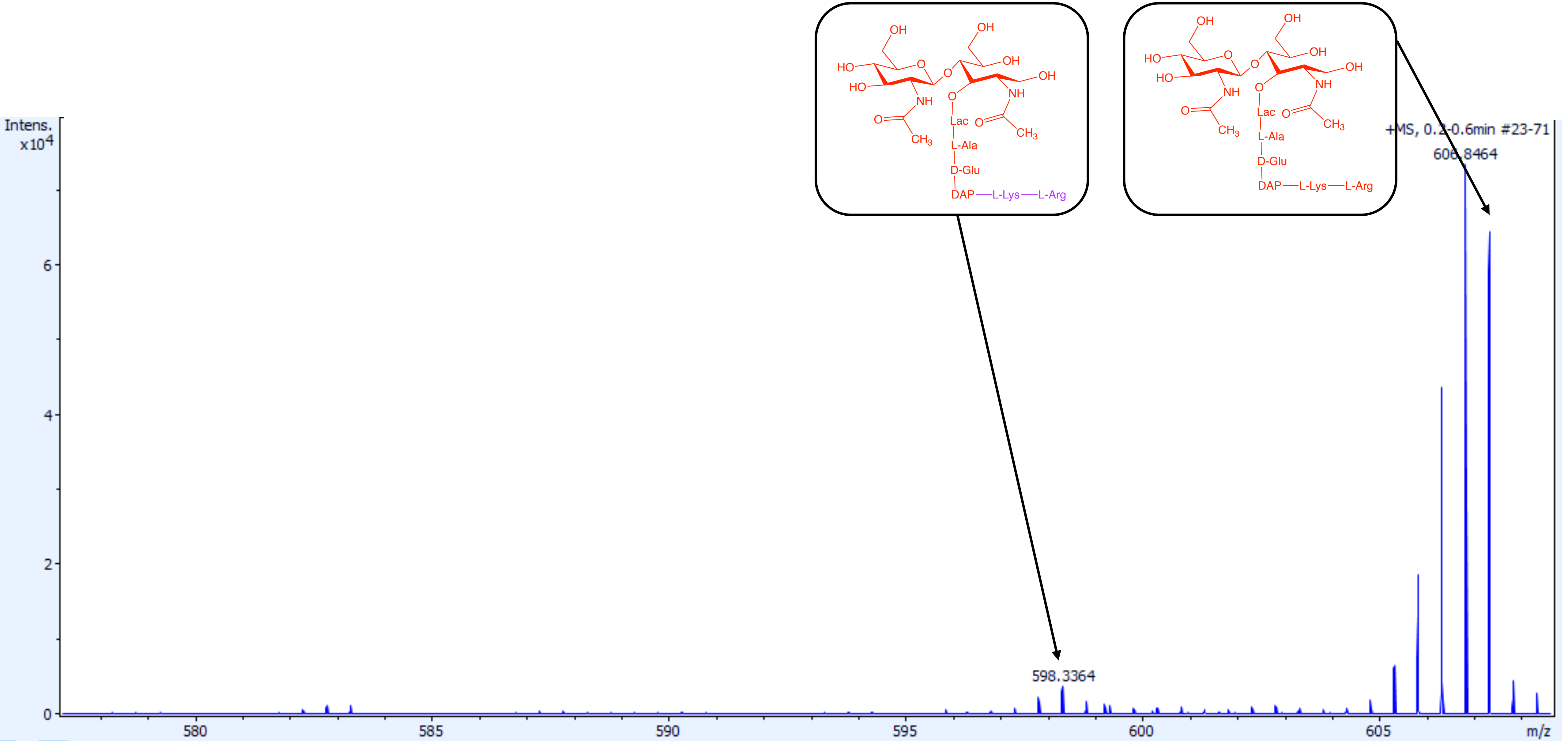

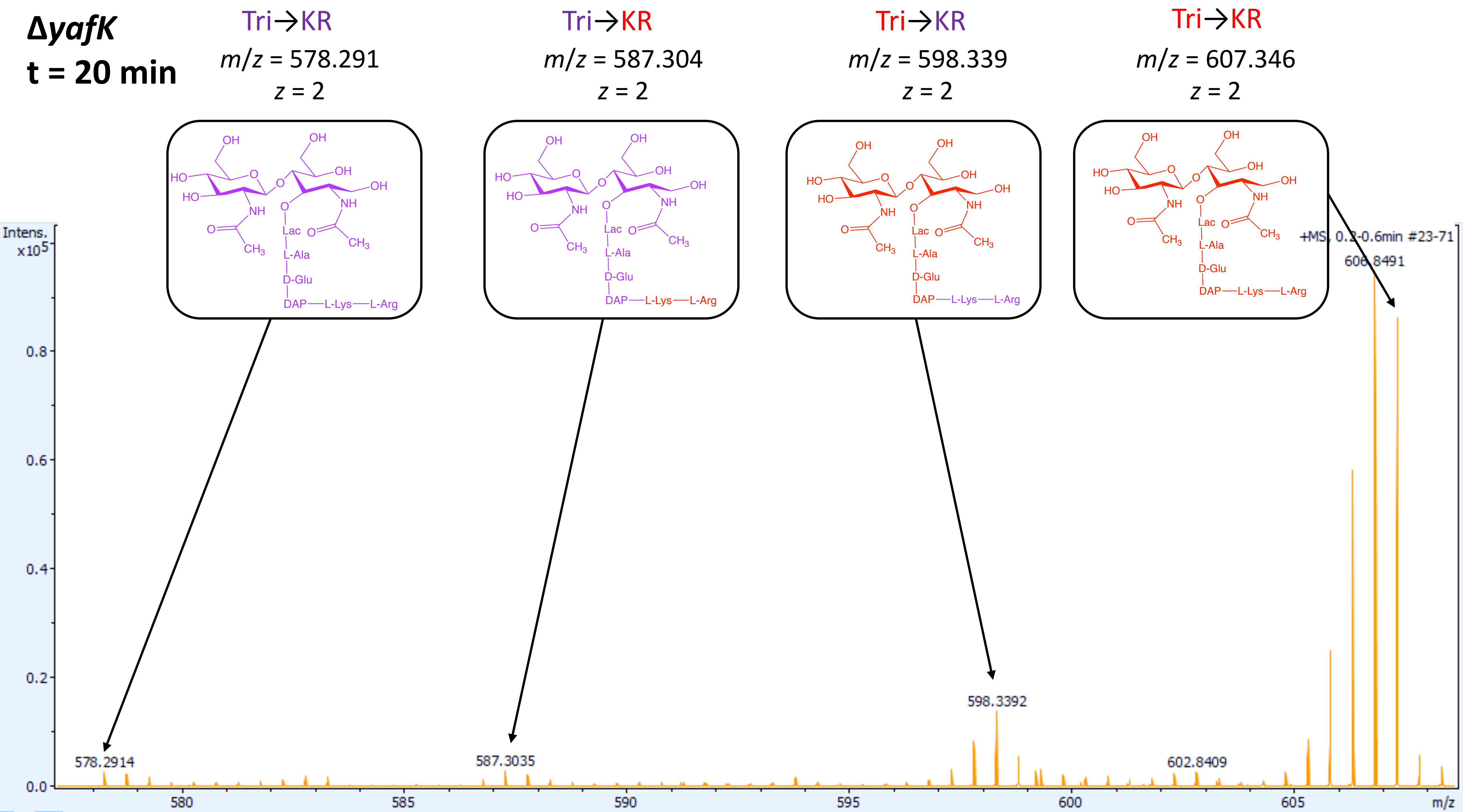

ΔyafK
t = 20 min
Tri→KR
m/z = 578.291
z = 2
Tri→KR
m/z = 587.304
z = 2
Tri→KR
m/z = 598.339
z = 2
Tri→KR
m/z = 607.346
z = 2
Lac
L-Ala
D-Glu
DAP—L-Lys—L-Arg
Intens.
x10^5
+MS, 0.2-0.6min #23-71
606.8491
598.3392
602.8409
587.3035
578.2914
m/z

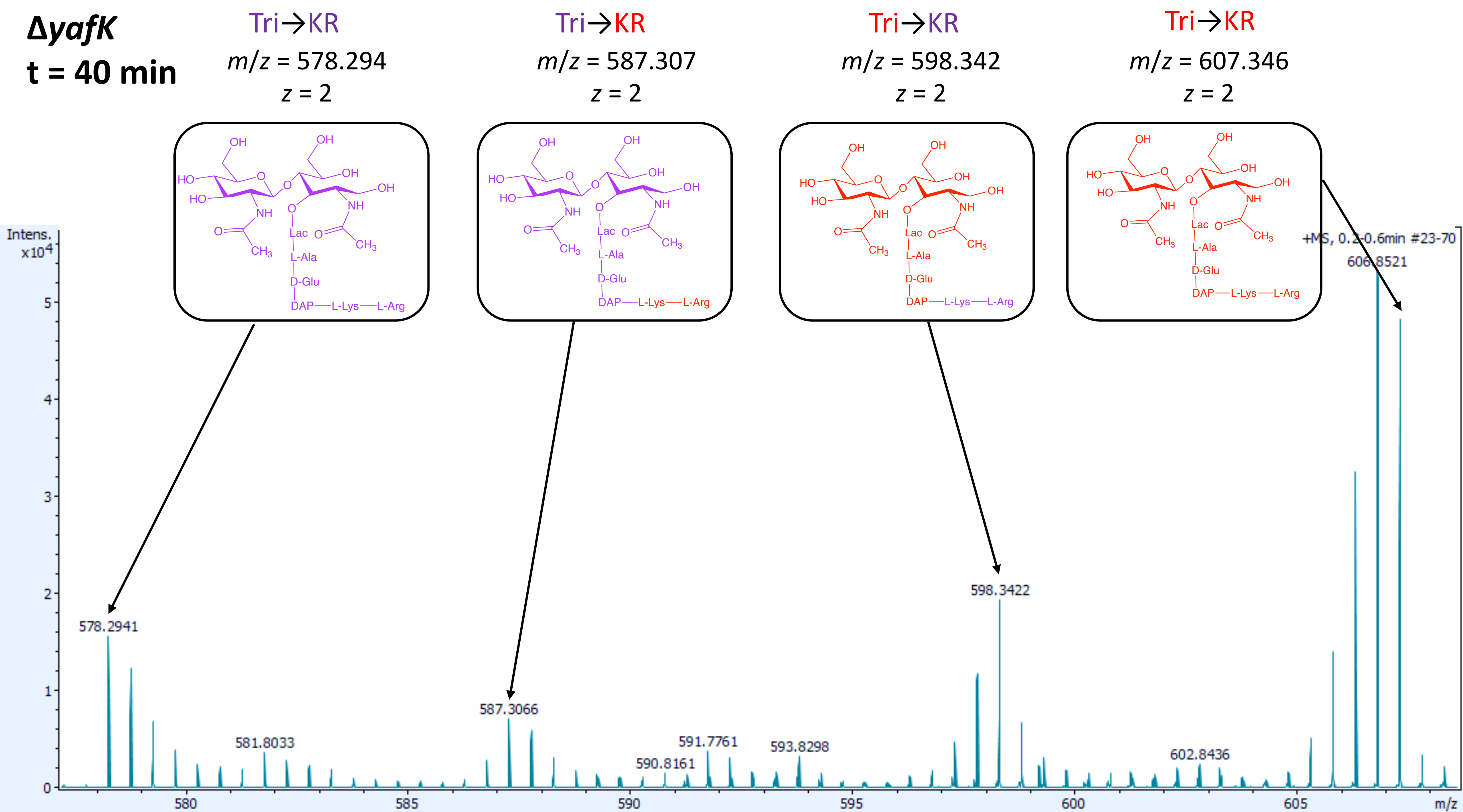

ΔyafK
t = 40 min
Tri→KR
m/z = 578.294
z = 2
Tri→KR
m/z = 587.307
z = 2
Tri→KR
m/z = 598.342
z = 2
Tri→KR
m/z = 607.346
z = 2
Lac
L-Ala
D-Glu
DAP—L-Lys—L-Arg
Intens.
x10^4
+MS, 0.2-0.6min #23-70
606.8521
598.3422
578.2941
587.3066
581.8033
590.8161
591.7761
593.8298
602.8436
m/z

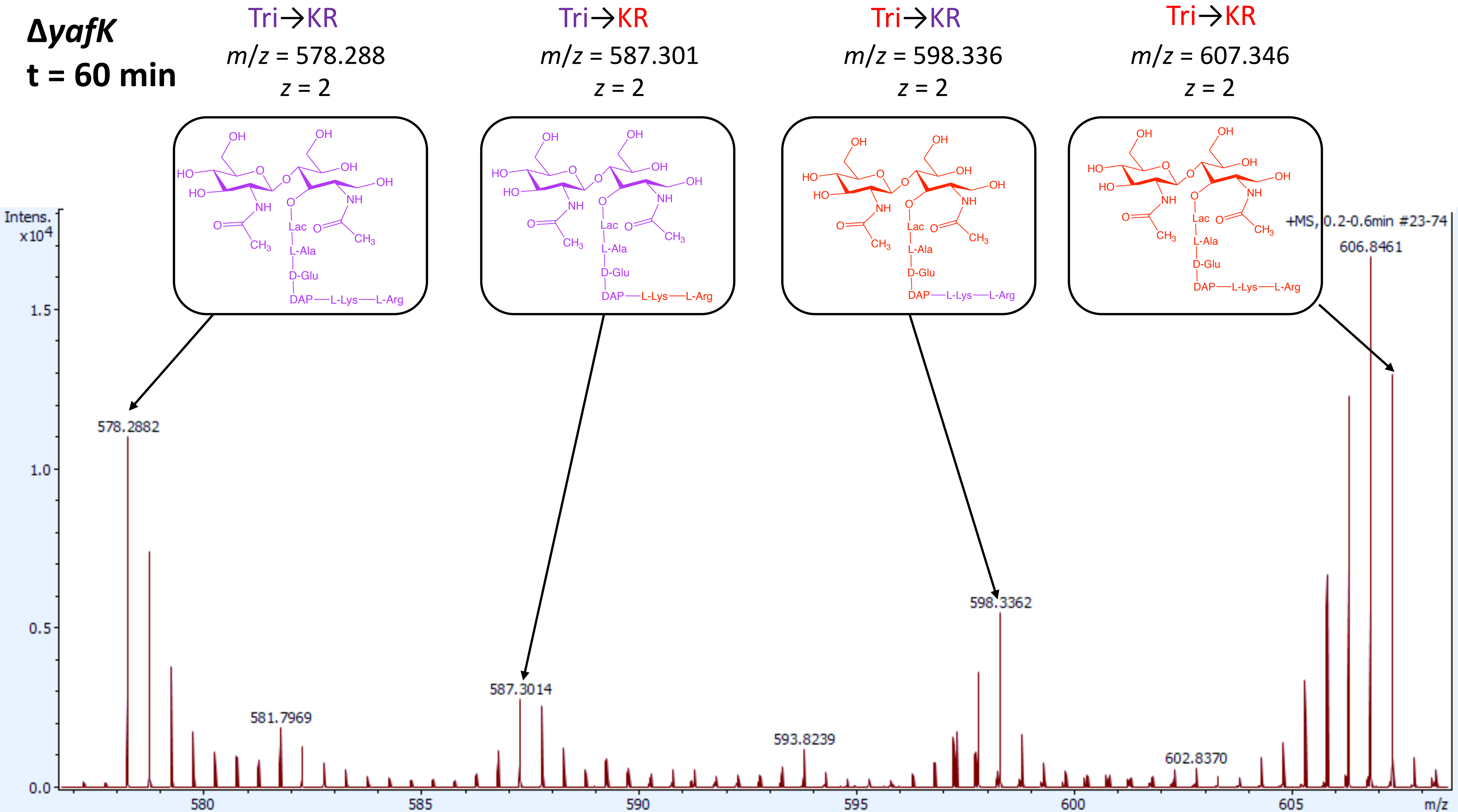

ΔyafK
t = 60 min
Tri→KR
m/z = 578.288
z = 2
Tri→KR
m/z = 587.301
z = 2
Tri→KR
m/z = 598.336
z = 2
Tri→KR
m/z = 607.346
z = 2
Lac
L-Ala
D-Glu
DAP—L-Lys—L-Arg
Intens. x10^4
+MS, 0.2-0.6min #23-74
578.2882
581.7969
587.3014
593.8239
598.3362
602.8370
606.8461
m/z
