# Supplementary material for: Peptidoglycan-tethered and free forms of the Braun lipoprotein are in dynamic equilibrium in Escherichia coli
Source: eLife. 2024 Oct 3;12:RP91598. doi: 10.7554/eLife.91598 (PMC11449479; doi:10.7554/eLife.91598)
Supplement: Supplementary file 3. [file elife-91598-supp3.docx]

| **Table S2. Kinetic analysis of the content of Tri➔KR isotopologues in new and old Tri and KR moieties** | | | | | | | | | | | | |
| --- | --- | --- | --- | --- | --- | --- | --- | --- | --- | --- | --- | --- |
|  |  | **Relative abundance (%) at 0, 10, 20, 40, and 60 min** | | | | | | | | | | |
| **Combinations of**  **Tri➔KR isotopologues** | **Biological**  **repeat** | **WT** | | | | |  | **Δ*yafK*** | | | | |
|  |  | **0** | **10** | **20** | **40** | **60** |  | **0** | **10** | **20** | **40** | **60** |
| Containing light KR  new➔new + old➔new | 1 | 0 | 6.57 | 14.68 | 31.38 | 47.85 |  | 0 | 6.17 | 15.74 | 38.71 | 58.45 |
|  | 2 | 0 | 5.23 | 12.15 | 33.03 | 50.53 |  | 0 | 3.57 | 8.92 | 25.92 | 46.25 |
|  | 3 | 0 | 3.21 | 10.39 | 43.32 | 53.95 |  | 0 | 3.46 | 10.38 | 26.81 | 55.20 |
|  | 4 |  | 4.95 |  |  |  |  |  |  |  | 29.48 |  |
|  | 5 |  | 3.73 |  |  |  |  |  |  |  |  |  |
|  | **Mean** | 0 | 4.74 | 12.41 | 35.91 | 50.77 |  | 0 | 4.40 | 11.68 | 29.93 | 53.30 |
|  | **SD** | 0 | 1.61 | 2.25 | 10.19 | 4.36 |  | 0 | 2.17 | 3.97 | 6.93 | 7.23 |
|  |  |  |  |  |  |  |  |  |  |  |  |  |
| Containing heavy KR  new➔old + old➔old | 1 | 100 | 93.43 | 85.32 | 68.62 | 52.15 |  | 100 | 93.83 | 84.26 | 61.29 | 41.55 |
|  | 2 | 100 | 94.77 | 87.85 | 66.97 | 49.47 |  | 100 | 96.39 | 89.47 | 73.89 | 53.75 |
|  | 3 | 100 | 96.79 | 89.61 | 56.68 | 46.05 |  | 100 | 96.65 | 89.53 | 76.52 | 44.80 |
|  | 4 |  | 95.05 |  |  |  |  |  |  |  | 70.52 |  |
|  | 5 |  | 96.27 |  |  |  |  |  |  |  |  |  |
|  | **Mean** | 100 | 95.26 | 87.59 | 64.09 | 49.23 |  | 100 | 95.62 | 87.75 | 70.56 | 46.70 |
|  | **SD** | 0 | 3.06 | 6.14 | 7.60 | 3.30 |  | 0 | 1.80 | 5.30 | 8.49 | 9.05 |
|  |  |  |  |  |  |  |  |  |  |  |  |  |
| Containing light Tri  new➔new + new➔old | 1 | 0 | 3.45 | 13.25 | 42.86 | 70.45 |  | 0 | 1.14 | 5.42 | 25.18 | 49.60 |
|  | 2 | 0 | 4.03 | 12.25 | 49.73 | 73.44 |  | 0 | 1.55 | 1.95 | 14.97 | 37.91 |
|  | 3 | 0 | 1.42 | 7.95 | 53.05 | 75.84 |  | 0 | 1.44 | 5.52 | 20.42 | 49.05 |
|  | 4 |  | 2.60 |  |  |  |  |  |  |  | 19.79 |  |
|  | 5 |  | 1.31 |  |  |  |  |  |  |  |  |  |
|  | **Mean** | 0 | 2.56 | 11.15 | 48.54 | 73.25 |  | 0 | 1.38 | 4.30 | 19.79 | 45.52 |
|  | **SD** | 0 | 1.29 | 2.82 | 12.59 | 4.86 |  | 0 | 0.50 | 2.04 | 6.37 | 6.66 |
|  |  |  |  |  |  |  |  |  |  |  |  |  |
| Containing heavy Tri  old➔new + old➔old | 1 | 100 | 96.55 | 86.75 | 57.14 | 29.55 |  | 100 | 98.86 | 94.58 | 74.82 | 50.40 |
|  | 2 | 100 | 95.97 | 87.75 | 50.27 | 26.56 |  | 100 | 98.41 | 96.44 | 84.84 | 62.09 |
|  | 3 | 100 | 98.58 | 92.05 | 46.95 | 24.16 |  | 100 | 98.67 | 94.39 | 82.92 | 50.95 |
|  | 4 |  | 97.40 |  |  |  |  |  |  |  | 80.21 |  |
|  | 5 |  | 98.69 |  |  |  |  |  |  |  |  |  |
|  | **Mean** | 100 | 97.44 | 88.85 | 51.46 | 26.75 |  | 100 | 98.65 | 95.13 | 80.70 | 54.48 |
|  | **SD** | 0 | 3.38 | 5.58 | 5.21 | 2.80 |  | 0 | 3.47 | 7.22 | 9.05 | 9.63 |
